# Green space exposure and active transportation during the COVID-19 pandemic: a global analysis using Apple mobility data

**DOI:** 10.1136/bmjgh-2024-017108

**Published:** 2025-05-14

**Authors:** Ruoyu Wang, Selin Akaraci, Esteban Moro, Pedro C Hallal, Rodrigo Reis, Ruth Hunter

**Affiliations:** 1 Centre for Public Health, QUB, Belfast, UK; 2 University of Essex, Colchester, UK; 3 UCL, London, UK; 4 Network Science Institute, Department of Physics, Northeastern University, Boston, Massachusetts, USA; 5 UIUC, Urbana, Illinois, USA; 6 People Health and Place Unit, School of Public Health Health, Washington University in St Louis, St Louis, Missouri, USA

**Keywords:** COVID-19, Global Health, Public Health

## Abstract

**Introduction:**

There is little evidence investigating the association between green space (exposure and inequality) and active transportation during the COVID-19 pandemic. This study focused on the spatial heterogeneity in trajectories of different transportation modes during the COVID-19 pandemic worldwide, as well as the association between green space exposure and inequality and active transportation during the COVID-19 pandemic from a global perspective.

**Methods:**

This study was based on an ecological study design and used three different Apple Mobility indices (driving, walking and public transit) to evaluate the trajectories of different transportation modes during the COVID-19 pandemic in 299 cities across 46 countries. Green space exposure was calculated based on fine-resolution population and green space mappings. Green space inequality was calculated by incorporating the Gini index into the green space exposure (green space Gini index). The hot/cold spot analysis was used to explore spatial heterogeneity in trajectories of different transportation modes during the COVID-19 pandemic worldwide, while Gaussian spatial mixed models were used to model the association between green space exposure and inequality and active transportation.

**Results:**

The hot/cold spot analysis shows that there were spatial inequalities in the trajectories of different transportation modes worldwide during the COVID-19 pandemic. Results from Gaussian spatial mixed models showed that green space exposure was positively associated with the walking index (Coef.=46.82; SE=18.20), while green space inequality was positively associated with the walking index (Coef.=58.88; SE=26.87) and public transit index (Coef.=162.07; SE=80.16). Also, the effect of green space varied across city development levels, the stringency of policy and COVID-19 severity.

**Conclusions:**

Our findings demonstrate the importance of sufficient city-scale green spaces to support active transportation, with important implications to help cities better prepare for future pandemics and support active transportation during non-pandemic times.

**WHAT IS ALREADY KNOWN ON THIS TOPIC**

**WHAT THIS STUDY ADDS**

- Apple mobility data can be used to evaluate different transportation modes (driving, walking and public transit) during the COVID-19 pandemic
- There was spatial heterogeneity in the trajectories of different transportation modes worldwide during the COVID-19 pandemic.
- The association between green space exposure and walking was positive during the COVID-19 pandemic, while the association between inequality in green space exposure and walking/public transit was also positive during the COVID-19 pandemic. Such associations vary across cities with different development levels.

**HOW THIS STUDY MIGHT AFFECT RESEARCH, PRACTICE OR POLICY**

- Our findings emphasise the need to enhance the availability of green space at the city level to encourage active transportation during the COVID-19 pandemic.
- Improving the provision of green space may especially help more deprived areas/countries to maintain active transportation.

## Introduction

 During the COVID-19 pandemic, many countries implemented several measures, including travel restrictions and social distancing measures, to reduce the transmission of the virus.^1^ This led to unprecedented restrictions on outdoor physical activity such as walking. During the pandemic, the intensity of city mobility declined worldwide, but not uniformly for all modes. For example, active transportation—such as walking and cycling—decreased more than motorised transportation.^2^ Active transportation promotes healthy environments and encourages physical activity,^3^,^5^ which has established health benefits.^6 7^ Existing evidence showed a large decline in global physical activity due to lockdown measures during the pandemic.^4 5^

Physical environments are increasingly recognised as crucial in facilitating active transportation.^8 9^ Among all physical environment factors, green space has attracted the greatest attention since promoting urban green space is recognised as a nature-based solution, proven to be an efficient strategy to achieve a wide range of Sustainable Development Goals and addressing social issues such as health and environmental issues simultaneously.^8 10^ Existing evidence has confirmed that higher green space provision is associated with more active transportation.^8^,^15^ Although many studies have also focused on inequality in green space provision,^16 17^ there has been little attention to inequality in green space provision and active transportation, especially during the COVID-19 pandemic. Higher inequality in green space provision may be negatively associated with active transportation. Typically, higher inequality in green space provision means more people share fewer green spaces. The increased crowdedness in green space may weaken its beneficial effects on human behaviours.^18 19^ On the other hand, higher inequality in green space exposure (ie, more green space for fewer people) may be positively associated with active transportation. Higher inequality in green space also means that the green space is sparser rather than concentrated in dense areas, so it is more likely to meet the demand of active transportation for different groups with various destinations.^20 21^

As shown in several reviews, there is a heterogeneous health/health behaviour influence of green space across different geographical contexts.^22^ For example, the ‘equigenesis’ theory suggests that green space may have more influence on socioeconomically deprived areas since green space is a kind of public infrastructure and it is freely available to vulnerable populations who are the majorities in socioeconomically deprived areas.^23^ Also, the impact of green space on human behaviour varies across different urbanicity levels, which is usually measured by the population density.^22^ While most of the studies did not find significant gradients in the effect of green space across different urbanicity levels,^24 25^ some studies showed that the green space–human behaviour associations seem to be stronger in more urbanised areas due to better maintenance of green spaces.^26^ During the pandemic, the severity level of COVID-19 may also have influenced green space-active transportation due to people’s fear of the virus and the shortage of front-line workers in the transportation sector.^27^ Furthermore, in response to the COVID-19 outbreak, many countries have implemented policy interventions on active transportation.^28^ As a result, the influence of physical environments on human behaviours may also vary depending on the intensity of the stringency of policy measures.^29^ This is because stronger stringency of policy can restrict people’s outdoor physical activities and the use of green spaces, which may mitigate the effect of natural environments.^29^

**Figure 1:**
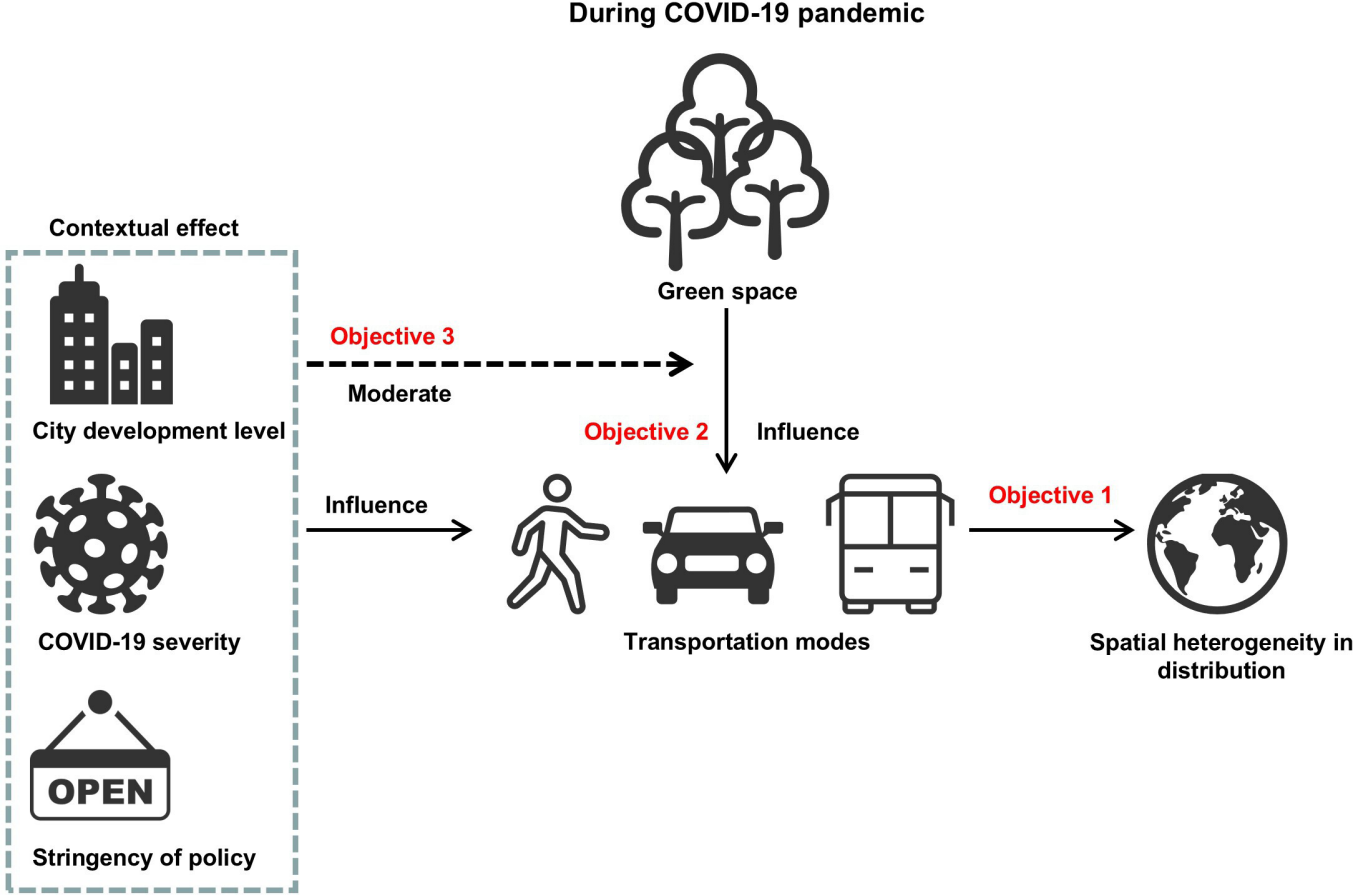
The theoretical framework.

In this study, we make a unique contribution to the current literature by analysing the trajectories of different transportation modes during the COVID-19 pandemic worldwide and the association with green space exposure and inequality with important implications for future pandemic preparedness and non-pandemic times. Specifically, our research objectives (figure 1) are to:

1. Investigate spatial heterogeneity in the trajectories of different transportation modes’ usage during the COVID-19 pandemic worldwide.
2. Examine how green space exposure and inequality are associated with active transportation during the COVID-19 pandemic.
3. Explore how the associations between green space and active transportation vary across various contexts, such as city development level, policy stringency and COVID-19 severity.

## Methods

### Study setting

For the analysis of transportation behaviour during COVID-19, we used Apple Mobility Trends Report data, which provided comparable data for 299 cities globally (online supplemental figure 1). While combining data from different mobile phone companies may improve the sample size, the timelines, systems and formats for these data are inconsistent. We limited our analysis to the city scale because the aggregated city-level data are the most fine-scale data provided by Apple to protect users’ privacy, and the alternative city scale is enough to accurately capture substantial heterogeneity in transportation, green space, sociodemographic and COVID-19 characteristics.

### Dependent variables

#### Apple Mobility Trends Report data

Transportation mode during the COVID-19 pandemic was from smartphones running Apple Mobility Map.^30^ Apple Mobility Trends Report data can be used to identify different transportation modes, including driving, walking and public transit.^30^ The data are aggregated at the level of the city to protect mobile users’ privacy.^30^ Apple Mobility Trends Report data are representative of people with smartphones since it is collected passively through the built-in application (map).^30^ Existing studies have found that Apple Mobility Trends Report data is highly correlated with official public transit and walking data across different geographies.^31^ One of the major advantages of using Apple Mobility Trends Report data is that it is collected in the same way and through the same technology in all geographies, which makes the data comparable worldwide. The driving, walking and public transit indices from Apple Mobility Trends Report data are available from January 2020 (baseline) to February 2022. As for each of the mobility indices, the baseline value is 100, and a value higher than 100 indicates the stronger intensity of the transportation mode, while a value lower than 100 indicates the weaker intensity of the transportation mode. The primary data are provided as daily records for 299 cities across 46 countries/regions. Since the daily records are relatively unstable, we aggregated the daily records into monthly records for all cities. Also, since most of the countries started to record COVID-19 cases in February 2020, we only included records from February 2020 (month 1) to December 2021 (month 23). The distribution of sampled countries is shown in online supplemental figure 1. All 299 cities were included in the spatial analysis. Also, after excluding cities with missing information, 224 cities were included in the final regression models. The flow diagram of the sample selection process is presented in online supplemental figure 2.

### Independent variables

We used two metrics to quantify availability and inequality in green space: green space exposure and green space Gini index provided by Chen *et al*.^17^

### Green space exposure

Green space exposure was measured by the population-weighted green space coverage rate.^17^ This was calculated by combining the WorldPop dataset^32^ and the European Space Agency’s (ESA) latest global baseline land cover product for 2020 (WorldCover) at a 10 m spatial resolution.^33^

The WorldPop dataset for 2020 was downloaded to quantify the spatial distribution of the population. WorldPop provides information regarding the number of populations in each 100×100 m grid. WorldCover data for 2020 was downloaded to quantify the pixel-level green space coverage rate. All types of forests, shrubs, grass, herbaceous wetlands and mangroves from the WorldCover maps were considered green spaces. The population-weighted exposure model proposed by Chen *et al*^17^ quantifies the spatial interaction between population and green space. It takes both population and green space into account, so it means higher weights were assigned to green space with more population. The details of the equation for calculating the population-weighted green space coverage rate are shown in the online supplemental notes 1–2 and figure 3. Population-weighted green space coverage rate values vary between 0 and 1. A higher value indicates more green spaces and a greener environment within the city boundary.

### Green space Gini index

The green space Gini index was measured by the Gini index in green space coverage rate.^17^ Chen *et al*^17^ proposed an algorithm to calculate the Gini index in green space coverage rate by combining the traditional Gini index framework and population-weighted green space coverage rate. The details of the equation for calculating the Gini index in green space coverage rate are shown in the online supplemental notes 3 and figure 4. The green space Gini index ranges from 0 to 1, with a higher value indicating that the amount of green space exposure is more equal within the city boundary. The calculation of city boundaries can also be found in the online supplemental note 4.

### Covariates

We controlled for a series of transportation-related, socioeconomic, demographic, environmental policy and pandemic-related variables as covariates following existing studies on the relationship between the built environment and travel behaviour during the pandemic.^34^,^36^ First, transportation-related variables were collected from Thompson *et al*.^37^ Cities were classified into eight types (high transit, chequerboard, informal, cul de sac, large block, irregular, intense and motor) based on a convolutional neural network and graph-based approach. These variables can reflect urban design characteristics within each city. Additionally, a series of dummy variables was included to control for seasonality. Second, demographic variables include sex (the number of males for every 100 females in a population) and age (median age), which were from The World Factbook (https://www.cia.gov/the-world-factbook/) and Population Division of the Department of Economic and Social Affairs of the United Nations (https://population.un.org/wpp/), respectively. Third, the socioeconomic status (SES)/level of each city was measured using the gross domestic product (GDP) per capita (US dollars), brightness of the night light data, and Human Development Index (HDI). GDP per capita measures the economic status of cities and was collected from the World Bank (https://www.worldbank.org/en/home) at the national level. Also, the brightness of the night light data was provided by the Earth Observation Group, which calculates the brightness using the global Visible Infrared Imaging Radiometer Suite (VIIRS) night-time lights.^38^ Such data can reflect the brightness of the night light and thus is usually treated as a proxy for the development level of the urban infrastructures. HDI from the United Nations Development Programme (https://hdr.undp.org/data-center/human-development-index#/indicies/HDI) was taken as a composite proxy for measuring the overall development level of each city. Fourth, population density (persons/km^2^) collected from the COVID-19 Open-Data platform^39^ was treated as the proxy for urbanity. Fifth, the annual concentration of particulate matter (PM_2.5_) (μg/m^3^) was provided by the Atmospheric Composition Analysis Group to measure air quality in each city.^40^ Sixth, policy and governance-related factors also play an important role during the pandemic,^29^ so we included the stringency index from The Oxford COVID-19 Government Response Tracker.^41^ These variables measure the strictness of ‘lockdown style’ policies that primarily restrict people’s outdoor behaviours.^29^ Lastly, the influence of the pandemic was measured by the COVID-19 severity in each city (the cumulative COVID-19 infection rate in 2020 and 2021), provided by the COVID-19 Open-Data platform.^39^ The details and operational definition of each covariate can be found in online supplemental table 1. Descriptive statistics of all variables are presented in online supplemental table 2.

### Statistical analysis

#### Hot spot analysis (Getis-Ord Gi*)

We applied the hot spot analysis (Getis-Ord Gi*)^42^ to investigate the spatial heterogeneity in trajectories of different transportation modes. The Getis-Ord Gi* function was proposed to calculate resultant z-scores and p values for each unit, which can further tell whether features with either high or low values cluster spatially. Finally, each unit was classified as either hot spots, cold spots or not significant. The details of such a method are shown in the online supplemental note 5.

### Gaussian spatial mixed models

To investigate the association between green space and different mobility indices, we used Gaussian spatial mixed models.^43^ The details of such a method are shown in the online supplemental note 6. The mean value of variance inflation factors (<4) suggested no severity of multicollinearity among predictors.

The results of the Gaussian spatial mixed model for driving, walking and transit were presented in model 1, model 2 and model 3, respectively. Furthermore, we conducted several moderation analyses to understand how the green space-mobility index associations vary across different contexts. First, we added the interaction term between green space exposure and green space Gini index to investigate how green space inequality may influence the effect of green space availability (model 4). Second, as suggested by the ‘equigenesis’ theory, green space may have a stronger effect on socioeconomically deprived places.^23^ We added the interaction term between green space metrics and GDP (model 5) as well as between green space metrics and HDI (model 6) to examine how the effect of green space varies across different SES levels. Third, existing evidence indicated that the function of green space may be influenced by urbanity level,^22^ so we also added the interaction term between green space metrics and population density (model 7) to test how the effect of green space varies across different urbanity levels. Fourth, governance and policy intervention may influence people’s outdoor activities during the pandemic,^29^ so we added the interaction term between green space metrics and stringency index (model 8) to test how the effect of green space varies across different intensity levels of governance. Last, previous studies suggested that the severity of the pandemic may have affected people’s behaviours,^27^ thus influencing the green space–transportation associations. Therefore, we added the interaction term between green space metrics and COVID-19 severity (model 9) to test how the effect of green space varies across different severity levels of the pandemic, which is important to understand the role of green space in the city’s resilience during the pandemic.

## Results

### The spatial heterogeneity in trajectories of different Apple mobility indices

The result for trajectories of different mobility indices from February 2020 to December 2021 (online supplemental figure 5) shows that the overall trajectories of different mobility indices were similar. They started to decline at the beginning of the pandemic until month 3 of the study period (April 2020). Although they tended to grow overall after month 3, they still fluctuated over that period. Turning points (ie, a point where the indexes change from sloping downwards to upwards) appeared in month 8 (November 2020), month 12 (January 2021) and month 20 (September 2021). Furthermore, the walking index was higher than the other two indices over the period, while the public transit index was the lowest.

Online supplemental figures 6–11 show the spatial distribution and clusters (see ‘Methods’ section) of the mobility indices (driving, walking and public transit) from 2020 to 2021, respectively. The hot spots (red circles in online supplemental figure 9–11) refer to units with high values of mobility indices and surrounded by high-value neighbours, while cold spots (blue circles in online supplemental figure 9–11) are the units with low values of mobility indices and surrounded by low-value neighbours. Both of them were considered as clusters, and identifying them can reveal how mobility indices during the pandemic are distributed unequally around the world and how such an inequality changes over time. First, the driving index was relatively high in North America and Japan (only Japan has a large number of observations as an Asian country) but low in most parts of Europe, South America, Oceania, Africa and most parts of Asia. The hot spot analysis (see ‘Methods’ section) indicated that hot spots were mainly distributed in North America and Japan, while the cold spots were mainly distributed across Europe. Hence, the number of hot/cold spots dropped throughout the period. Second, the walking index was relatively high in North America, some parts of Europe (eg, West Europe), and Japan but low in South America, Oceania, Africa and most parts of Asia. In the early stage of the pandemic (before February 2021), the hot spots were mainly in North America, Europe and Japan. At the late stage of the period, the hot spots were still mainly in North America, but the cold spots were mainly found in Japan. Hence, there were not many significant spots in Europe at the late stage of the period. Last, the public transit index was high in Europe but low in other continents. The hot spots in the early period were mainly detected in Europe and Japan, while the cold spots were found in North America. Although the hot spots were still mainly located in Europe in the late period, there were not many significant spots in North America and Japan.

### Association between green space (exposure and inequality) and the different Apple mobility index

**Figure 2:**
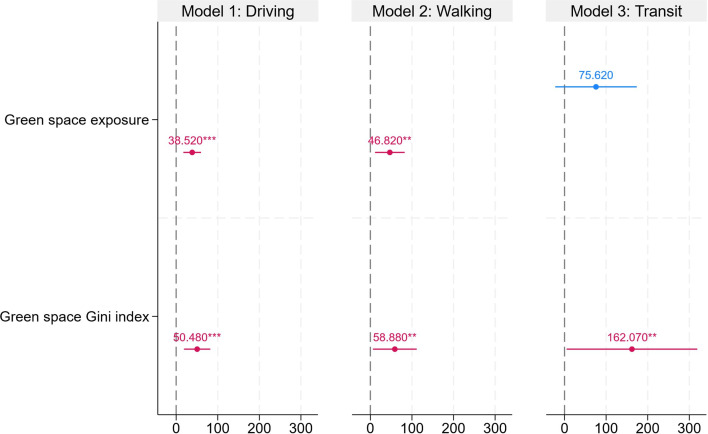
Regression results for the mixed effect model to examine the association between green space exposure and inequality and Apple mobility index. All covariates were adjusted. The bars indicate the 95% CIs. **p<0.05, ***p<0.01. The full models can be found in online supplemental table 3.

Figure 2 and online supplemental table 3 present the association between green space (exposure and inequality) and different Apple mobility indices during the COVID-19. The result (model 1) showed that both green space exposure (Coef.=38.52; SE=10.84) and green space Gini index (Coef.=50.48; SE=16.14) were positively associated with the driving index. Also, model 2 indicated that both green space exposure (Coef.=46.82; SE=18.20) and green space Gini index (Coef.=58.88; SE=26.87) were positively associated with the walking index. Furthermore, model 3 showed that there is no evidence that green space exposure is associated with the public transit index (Coef.=75.62; SE=49.99), while the green space Gini index was positively linked to the public transit index (Coef.=162.07; SE=80.16).

**Figure 3:**
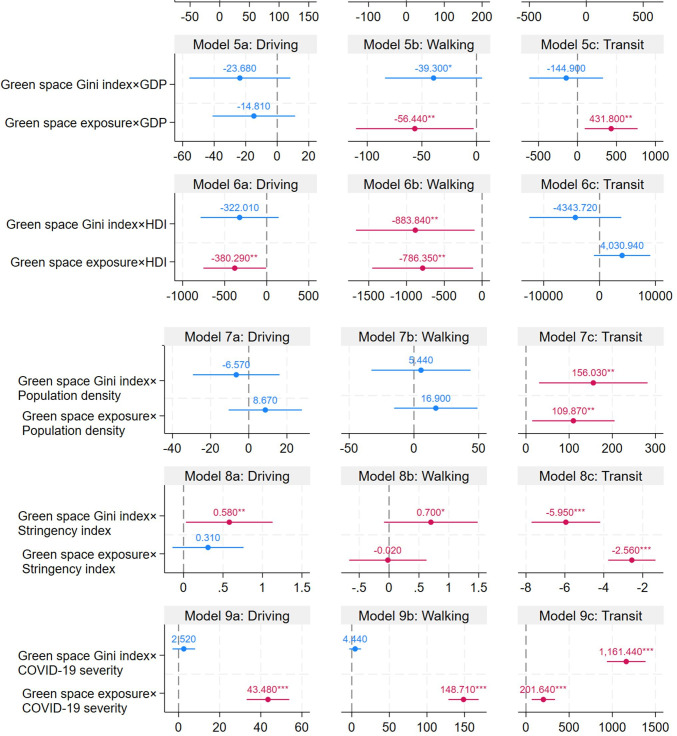
Regression results for the mixed effects model to examine the association between green space exposure and inequality, and Apple mobility index (moderation analysis). All covariates were adjusted. The bars indicate the 95% CIs. *p<0.10, **p<0.05, ***p<0.01. The full models can be found in online supplemental table 4. GDP, gross domestic product; HDI, Human Development Index.

The moderation analysis is displayed in figure 3 and online supplemental table 4. Model 5b and online supplemental figure 12a show that GDP negatively moderated the association between green space exposure and the walking index (Coef.=−56.44; SE=27.46). Also, Model 5c and online supplemental figure 12a show that GDP positively moderated the association between green space exposure and the public transit index (Coef.=431.80; SE=173.09). Model 6a and online supplemental figure 12b suggest that HDI negatively moderated the association between green space exposure and the driving index (Coef.=−380.29; SE=190.51), while model 6b and online supplemental figure 12b present that HDI negatively moderated the green space exposure-walking index association (Coef.=−786.35; SE=340.60) as well as green space inequality-walking index association (Coef.=−883.84; SE=400.40). Model 7c and online supplemental figure 12c indicate that population density positively moderated the green space Gini index—public transit index association (Coef.=156.03; SE=64.17) and green space exposure—public transit index association (Coef.=109.87; SE=48.70). While model 8a and online supplemental figure 12d show that the stringency index positively moderated the relationship between green space Gini index and driving index (Coef.=0.58; SE=0.28), model 8c and online supplemental figure 12d indicate that stringency index negatively moderated the green space Gini index—public transit index association (Coef.=−5.95; SE=0.90) as well as green space exposure—public transit index association (Coef.=−2.56; SE=0.62). Furthermore, model 9 and online supplemental figure 12e suggest that COVID-19 severity positively moderated the green space Gini index—public transit index association (Coef.=1161.44; SE=114.27), green space exposure—driving index association (Coef.=43.48; SE=5.28), green space exposure—walking index association (Coef.=148.71; SE=10.28) and green space exposure—public transit index association (Coef.=201.64; SE=68.94).

## Discussion

Using Apple Mobility Trends Report data, we showed that there is spatial heterogeneity in the trajectories of different transportation modes during the pandemic. Also, the association between green space and active transportation was positive during the COVID-19 pandemic, which indicates green space may play an important role in such a period. We further found that such an association varied across city development levels, stringency of policy and COVID-19 severity. Our results demonstrated that although both green space exposure and inequality may contribute to active transportation during the COVID-19 pandemic, their effects are conditional and contextual. This demonstrates the importance of sufficient city-scale green spaces to support active transportation, with important implications to help cities better prepare for future pandemics and support active transportation during non-pandemic times.

### Spatial heterogeneity in trajectories of different transportation modes

Although there was a decline for all transportation modes (driving, walking and public transit) at the beginning of the pandemic, they recovered in a few months, and, in some cases, all increased beyond prepandemic levels. This finding is consistent with existing evidence from different continents.^3144^,^46^ A possible explanation is that the policy intervention on mobility restriction was relatively strict in the early phase of the pandemic but then reduced as time passed due to economic recession.^28^ Since most Apple Mobility Trends Report data are collected from high-income countries (HICs), the spatial characteristics of different mobility indices are more significant in HICs. Overall, the driving index and the walking index were high in North America, while the public transit index was high in Europe. This may be because car ownership is especially high in North American countries such as America and Canada,^47^ while public transit systems are more developed in Europe.^48^ We also found that spatial clusters of different mobility indices were decreasing, which means the distribution of mobility indices has become more equal worldwide as the inequality declines from the early to the late phase of the pandemic. This may provide evidence for the existence of city resilience for mobility, which means the more the cities were affected by COVID-19 at the early stage, the greater capacity they had to recover at the later phase of the pandemic.

### The association between green space exposure and active transportation

Our results suggested that green space exposure was associated with more active transportation. Specifically, green space exposure was positively associated with the walking index during the COVID-19 pandemic. There are several explanations for such a finding. First, previous studies have found that exposure to green spaces is negatively associated with COVID-19 case rates, as green spaces may strengthen the human immune system.^49^ Therefore, individuals living in greener areas may feel less susceptible to virus transmission and are more willing to venture outside during the pandemic.^16^ Additionally, green space offers an ideal environment for social interaction during the pandemic since it allows for effective social distancing.^50^ Thus, green spaces may play a crucial role in promoting recreational walking and the use of public transit during the pandemic, aligning with existing findings.^44^ Second, the restorative effect of green spaces may also partly explain our findings. For example, some studies have shown that people are more likely to choose greener routes for walking or running because it helps distract them from daily stress.^51^,^53^ This finding is also confirmed in driving behaviour, where greener environments positively impact drivers’ mental states.^14^ More importantly, the pandemic has dramatically worsened mental well-being and increased the burden of mental illness,^54^ leading travellers to seek environments with better restorative functions.

### The association between green space inequality and active transportation

The results also suggested that the green space Gini index was positively associated with the walking index and public transit index during the COVID-19 pandemic. One possible explanation is that although higher inequality in green space provision means a small number of people may have better access to more green spaces, it is still possible that these populations happen to have a higher demand for or simply have to travel outside during the COVID-19 pandemic.^55^ Another possible explanation is that most of the sampled cities are from developed countries where the green space Gini index is relatively low, so a higher Gini index in green space provision may also indicate a sparser distribution of green space in urban areas. Existing studies pointed out that green spaces in multiple contexts (eg, workplace and recreational places) and destinations matter for people’s transportation behaviour.^56^ Therefore, when the distribution of green space is sparser, it is more likely that green space can cover more contexts and destinations throughout the city, thus facilitating more travel.

### Disparities in the association between green space and active transportation across cities

The moderation analysis confirmed that the green space-transportation associations are conditional on different contexts. First, the green space-walking index association was stronger for cities with lower GDP. The ‘equigenesis’ theory indicates that socioeconomically deprived areas are more influenced by public infrastructures such as urban parks since they are freely available resources.^23^ During the pandemic, the socioeconomically deprived areas were even more vulnerable than before since most resources were used to stop the transmission of the virus.^57^ Therefore, our findings may imply that green space could play a more crucial role in maintaining active travel for low-income and middle-income countries (LMICs). However, the green space—public transit index association was stronger for cities with higher GDP. This may be because cities with higher GDP are more likely to have better public transit infrastructure, system and service,^58^ so they can better function during the pandemic. Second, the green space-driving index association and green space-walking index association were stronger for cities with lower HDI. This, again, is consistent with the ‘equigenesis’, which suggests that socioeconomically deprived areas may be influenced more by the green space.^23^ Third, the green space—public transit index association was stronger for cities with higher population density. Public transit stations are denser and more developed in more urbanised cities,^59^ so they may better support public transit travellers and strengthen the effect of green space during the pandemic. Fourth, the green space–public transit index association was stronger for cities with a lower stringency index. Less intense stringency of the policy usually means less restriction on public transit and outdoor activities,^60^ and people are more likely to even consider travelling by public transit under such circumstances. Lastly, the green space-walking and green space-public transit index associations were stronger for cities with higher levels of COVID-19 severity. During the pandemic, the higher the COVID-19 severity, the more social distance may be needed for travelling,^60^ so green space can act as a buffer and contribute to outdoor travel (especially active travel such as walking and public transit). Existing evidence has suggested that COVID-19 has posed great mental stress on people’s daily lives,^54^ so travellers are more likely to be in higher demand for more restorative scenes provided by green space when the level of COVID-19 severity is high.

### Strengths and limitations

There are significant strengths for our study. First, Apple Mobility Trends Report data were collected from users worldwide,^44^ which ensures we can understand the association between green space exposure and inequality, and active transportation during the COVID-19 pandemic from a global perspective. Also, Apple Mobility Trends Report data were collected over a long period, so our analysis was based on a longitudinal study design. Second, Apple Mobility Trends Report data can be decomposed into three different transportation modes (driving, walking and public transit),^44^ so we can further compare the results of different transportation modes. Third, when calculating green space exposure, it was weighted by population, which makes it more accurate for reflecting population-level exposure.^17^ Hence, not only did we include green space exposure, but also, we also considered inequality in green space exposure, which makes the green space metric more comprehensive.

Our study also has some limitations. First, although the proportion of mobile phone users is increasing, there are significant differences across geographies, and even within cities and countries.^44^ Apple ownership is more prevalent among HICs, and the use of Apple Maps might be more frequent in certain places rather than evenly distributed.^44^ Thus, only a small amount of the Apple Mobility Trends Report data is from LMICs, which means a full understanding of the spatial heterogeneity in transportation modes during the pandemic cannot be fully investigated. Hence, the Apple Mobility index might be biased towards LMICS, some specific mobility behaviours, and LMICs.^44^ Future studies should consider using more sources of data from LMICs to overcome the bias. Second, the green space data was collected only for 2020, which prevents us from inferring how the changes in green space exposure influence transportation modes. Also, the exposure to green space was calculated using the ESA WorldCover data, which does not indicate whether the green space is publicly accessible. Consequently, the measurement of green space exposure could be skewed if most of the ESA-based green space in a specific city is not accessible. Third, we used a categorical variable as the proxy for transportation-related infrastructures, which may not be precise enough to measure the variances in transportation-related infrastructures across geographies. Also, GDP and HDI were measured at the national level, which may not be able to reflect the actual differences between cities. Therefore, the economic level may be underestimated in those global cities (eg, New York and London), but overestimated in those underdeveloped cities. Fourth, this study was based on an ecological study design, which may lead to ecological fallacy.^61 62^ Therefore, the findings from this study may not be valid for individuals and thus reduce the generalisability of our results. Lastly, this study may also be affected by selection bias due to unmeasured variables. For instance, weather conditions and unobserved urban planning policies can impact green space exposure and transportation modes. Also, the lack of sociodemographic information on iPhone users prevents us from conducting subgroup analysis.

## Conclusions

Our results indicated that there was spatial heterogeneity in the trajectories of different transportation modes. Also, both green space exposure and inequality in green space exposure were positively associated with active transportation during the pandemic. This study further suggested that green space-active transportation associations varied by city development level, stringency of policy, and COVID-19 severity. Our findings highlight the necessity of building sufficient green infrastructure at the city scale in promoting active transportation during the COVID-19 pandemic. This means improving the provision of green space may help the world (especially for more deprived areas/countries) in non-pandemic times and also better prepare for the next pandemic.

## Supplementary material

10.1136/bmjgh-2024-017108 online supplemental file 1

## Data Availability

Data are available in a public, open access repository. Data may be obtained from a third party and are not publicly available.
