# Supplementary material for: Green space exposure and active transportation during the COVID-19 pandemic: a global analysis using Apple mobility data
Source: BMJ Glob Health. 2025 May 14;10(5):e017108. doi: 10.1136/bmjgh-2024-017108 (PMC12083287; doi:10.1136/bmjgh-2024-017108)
Supplement: online supplemental file 1 [file bmjgh-10-5-s001.pdf]

Supplementary Information

Green space exposure and active transportation during the COVID-19 pandemic: A global analysis using Apple mobility data

Ruoyu Wang, Selin Akaraci, Esteban Moro, Pedro C Hallal, Rodrigo Reis, Ruth F. Hunter

Supplementary Notes

Supplementary Note 1 Pixel-level green space coverage rate 1
Supplementary Note 2 Population-weighted green space coverage rate 1
Supplementary Note 3 Gini index in green space coverage rate 2
Supplementary Note 4 The calculation of city boundary 2
Supplementary Note 5 Hot Spot Analysis (Getis-Ord Gi*) 2
Supplementary Note 6 Gaussian spatial mixed models 3

Supplementary Figures

Supplementary Fig S1 4
Supplementary Fig S2 4
Supplementary Fig S3 5
Supplementary Fig S4 5
Supplementary Fig S5 6
Supplementary Fig S6 7
Supplementary Fig S7 8
Supplementary Fig S8 9
Supplementary Fig S9 10
Supplementary Fig S10 11
Supplementary Fig S11 12
Supplementary Fig S12 13

Supplementary Tables

Supplementary Table S1 15
Supplementary Table S2 17
Supplementary Table S3 17
Supplementary Table S4 18

Supplementary Note 1 Pixel-level green space coverage rate

The 10-m WorldCover green space binary map was aggregated into 100-m grids to match the 100-m WorldPop population data. Then, pixel-level green space coverage rate we calculated for each 100-m pixel according to Eq. (S1):

$$G = \frac{\sum_{i=1}^{n} g_i}{n} \quad (S1)$$

where g_i represents the presence of green space (10-m WorldCover green space binary map) of the i-th grid, n is the total number of grids within each of the 100-m grids, and G is the pixel-level green space coverage rate.

Supplementary Note 2 Population-weighted green space coverage rate

$$GE = \frac{\sum_{i=1}^{n} P_i \times G_i}{\sum_{i=1}^{n} P_i} \quad (S2)$$

where P_i represents the population of the i-th grid, G_i represents the green space coverage rate of the i-th grid considering nearby green environments with a buffered radius of 500 m (Fig S4), n is the total number of grids within the corresponding city boundary, and GE is the corresponding population-weighted green space coverage rate level.

Supplementary Note 3 Gini index in green space coverage rate

As shown in Fig. S5 Gini index is mathematically calculated as the ratio of the area that lies between the line of equality and the Lorenz curve (region A) over the total area under the line of equality (region A + region B). therefore existing studies[1] have summarized the Gini index in green space coverage rate as Eq. (S3):

$$\mathrm{Gini} = 1 - \frac{\sum_{i=1}^{n}\sum_{j=1}^{i-1} g_j + \sum_{i=1}^{n}\sum_{j=1}^{i} g_j}{n \times \sum_{j=1}^{n} g_j}$$

(S3)

where g_j is the green space coverage rate for the j-th resident, and n is the total resident number within the city. Also, i represents the i-th part of region B.

Supplementary Note 4 The calculation of city boundary

We used the Global Urban Boundaries data[3], which represents the built-up areas corresponding to each of the sampled cities. This data was generated based on the Landsat imagery using a hierarchical approach, which includes the built-up areas of both city centres fringes[3].

Supplementary Note 5 Hot Spot Analysis (Getis-Ord Gi*)

The Getis-Ord Gi* function[3] was proposed to calculate resultant z-scores and p-values for each unit, which can further tell whether features with either high or low values cluster spatially. Finally, each unit was classified as either hot spots, cold spots, or not significant. The hot spots refer to units with high values and surrounded by high-value neighbours, while cold spots are the units with low values and surrounded by low-value neighbours. Therefore, A hot or cold spot is a unit showing statistically significantly high or low values of incidents[3]. Both hot and cold spots were considered clusters, which are a series of adjacent geographical units sharing a similar character (either in high or low values of mobility indices). The above process was calculated in ArcGIS 10.8 (Esri, Redlands, CA) with the function of 'Hot Spot Analysis (Getis-Ord Gi*). To display how the distribution of hot/cold spots changes over time, we presented the distribution of hot/cold spots every four months (February, June, and October) over two years (2020 and 2021). This can help us identify whether and how mobility indices during the pandemic are distributed unequally around the world and how such an inequality changes over time.

$$G_i^* = \frac{\sum_{j=1}^{n} w_{i,j}x_j - \bar{X}\sum_{j=1}^{n} w_{i,j}}{\sqrt{\frac{\sum_{j=1}^{n} x_j^2}{n} - (\bar{X})^2}\sqrt{\frac{[n\sum_{j=1}^{n} w_{i,j}^2 - (\sum_{j=1}^{n} w_{i,j})^2]}{n-1}}}$$

(S4)

where G_i^* is the Getis-Ord Gi* of city *i*; n is the total number of cities; x_i is the Apple

mobility index of city *i*; x_j is the Apple mobility index of city *j* (*i* ≠*j*); $\bar{X}$ is the mean value of Apple mobility index from all cities; $w_{i,j}$ is the spatial matrix and is equal to the reciprocal of the distance between city *i* and city *j* (inverse distance weights). G_i^* is a z-score, so if it is > 0 and its corresponding p-value is significant under 95% level, then city *i* will be defined as a hot spot; if it is < 0 and its corresponding p-value is significant under 95% level, then city *i* will be defined as a cold spot.

Supplementary Note 6 Gaussian spatial mixed models

When spatial dependence of mobility index exists, the application of OLS (ordinary least squares) models cannot give accurate estimations. As Moran's I[4] of all three indexers was positive and significant, it is necessary to consider the effect of spatial dependence. Some existing studies have tried to use eigenvector spatial filtering (ESF) to eliminate the influence of spatial dependence[4]. ESF is like a spatial weight matrix that defines the spatial associations among different units using the weighted sum of the Moran eigenvectors (MEs) [4]. After adding the ESF term to the model, the spatial dependence of the mobility index can be efficiently eliminated. Also, a mixed effect model approach was necessary due to the hierarchical structure of the data, as measurements at each month were nested within cities (our primary geographical unit), and cities were nested within countries. The intra-class correlation coefficient (ICC) for the null model predicting the driving index is 0.46 at the country level and 0.06 at the individual city level, respectively; The ICC for the null model predicting the walking index is 0.32 at the country level and 0.15 at the individual city level, respectively; The ICC for the null model predicting public transit index is 0.30 at the country level and 0.20 at the individual city level, respectively. This means that locating in the same country accounted for 46% of the total variation in the driving index, 32% of the total variation in the walking index, and 30% of the total variation in the public transit index, respectively. Hence, individual city-level variation accounted for 6% of the total variation in the driving index, 15% of the total variation in the walking index, and 20% of the total variation in the public transit index, respectively. Such results confirmed the necessity of mixed-effect models. Gaussian spatial mixed models combine both ESF and mixed effect model approach[4]. The final model was estimated as follows:

$$y_{tij} = \beta_0 + \beta_1 x_{tij} + f_{MC}(s_i) + \varepsilon_{tij} + \mu_{ij} + \varphi_j \tag{S5}$$

Where *t* represents time (month), *i* represents individual cities, and j represents countries. x_{tij} represents a vector of predictors (green space and covariates); $f_{MC}(s_i)$is the ESF depending on the location s_i of city *i* (coordinates); ε_{tij}, μ_{ij} and φ_j represent random errors within individual cities, between individual cities, and between countries, respectively.

Reference

1 Chen, B. *et al.* Contrasting inequality in human exposure to greenspace between cities of Global North and Global South. *Nature Communications* **13**, 4636 (2022).

2 Getis, A. & Ord, J. K. The analysis of spatial association by use of distance statistics. *Geographical analysis* **24**, 189-206 (1992).

3 Li, X. *et al*. Mapping global urban boundaries from the global artificial impervious area (GAIA) data. *Environmental Research Letters* **15**, 094044 (2020).

4 Murakami, D. Spatial regression modelling using the spmoran package: Boston housing price data examples. *arXiv preprint arXiv:1703.04467* (2017).

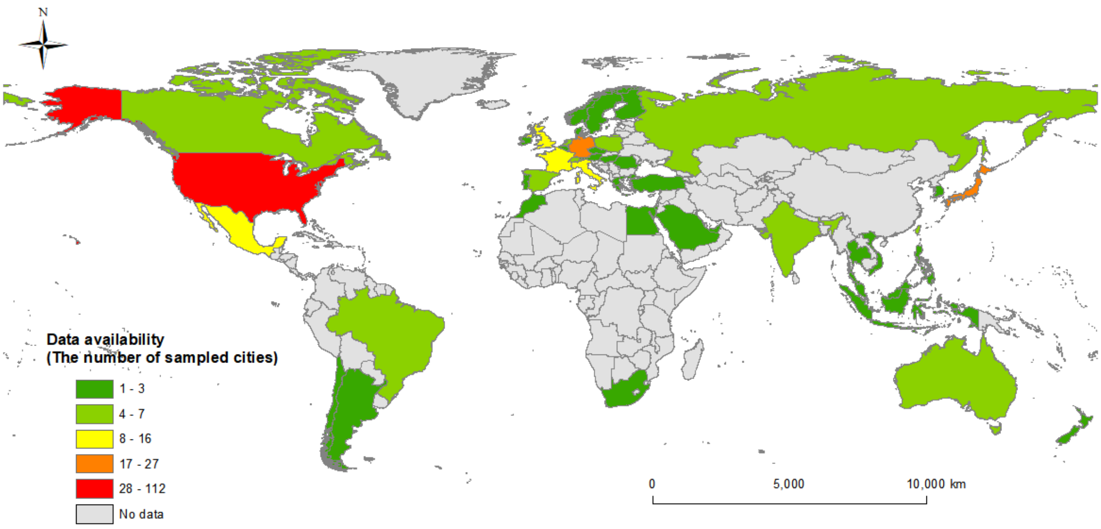

Fig. S1 The distribution of sampled countries/regions

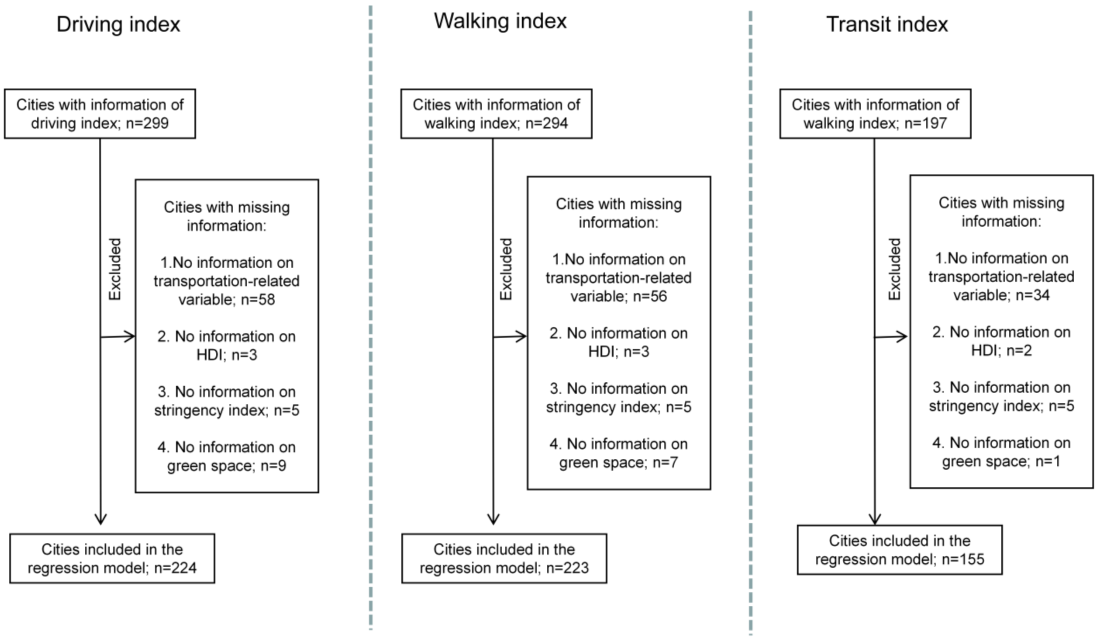

Fig S2: Flow diagram of the sample selection process

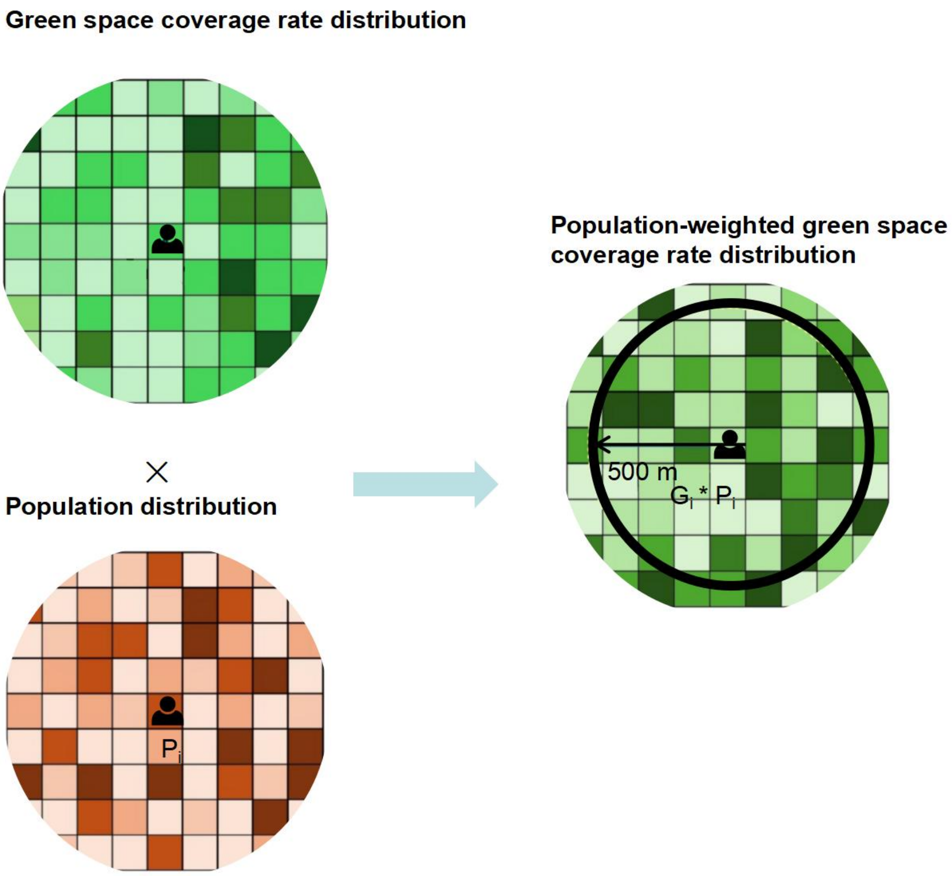

Figure S3. Conceptual diagram of population-weighted green space coverage rate within a 500-m buffer distance from human settlements. The population-weighted measure provides proportionally greater weight to green space near areas of higher population density. The buffer distance is calculated from the grid centre, and any grid within the dashed buffer was included in the calculation.

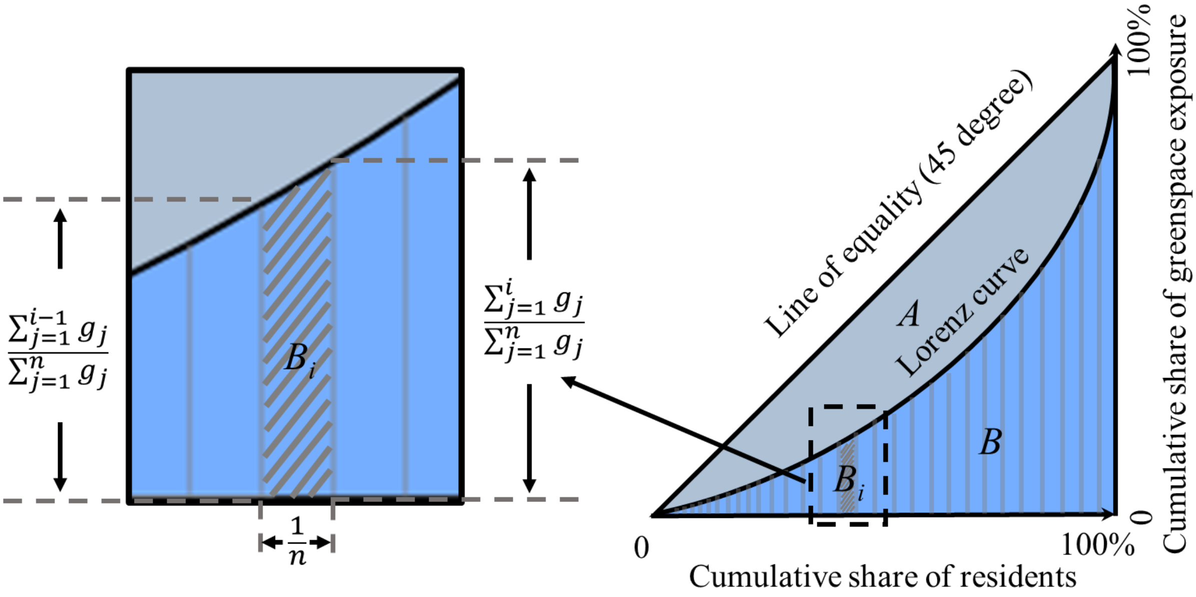

(a) (b)

Figure S4. Illustrative diagram of Gini index-based inequality assessments of green space coverage rate. The Gini index is defined as the ratio of the area that lies between the line of equality and the Lorenz curve (region A) over the total area under the line of equality (region A plus region B). Lorenz curve plots the proportion of the green space coverage rate (y-axis) that is cumulatively resided by the residents within a certain city (x-axis). This figure is adapted from Chen et al. (2022), and used under CC BY 4.0. It is licensed under CC BY 4.0 by [Ruoyu Wang]

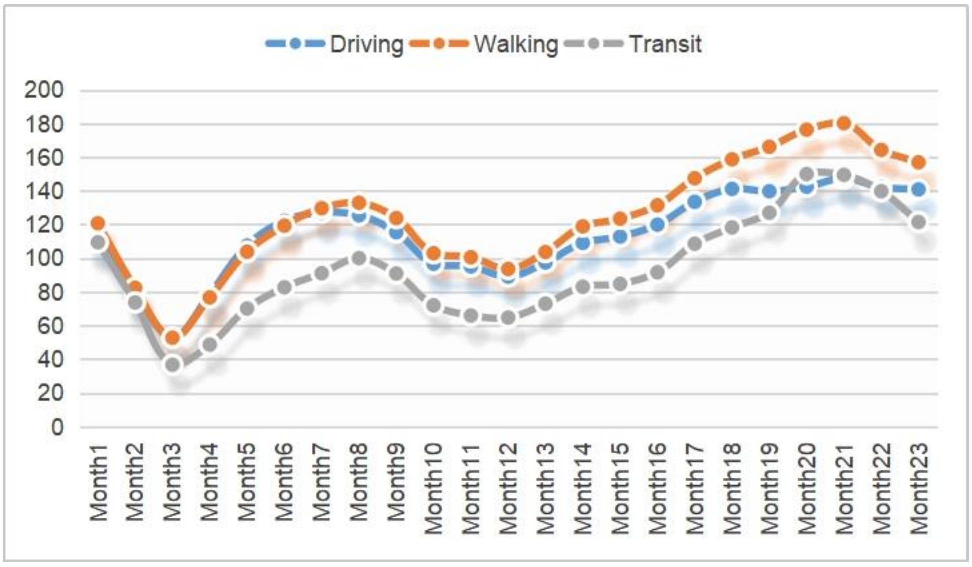

Fig. S5 The trajectories of different Apple mobility indices from February 2020 to December 2021

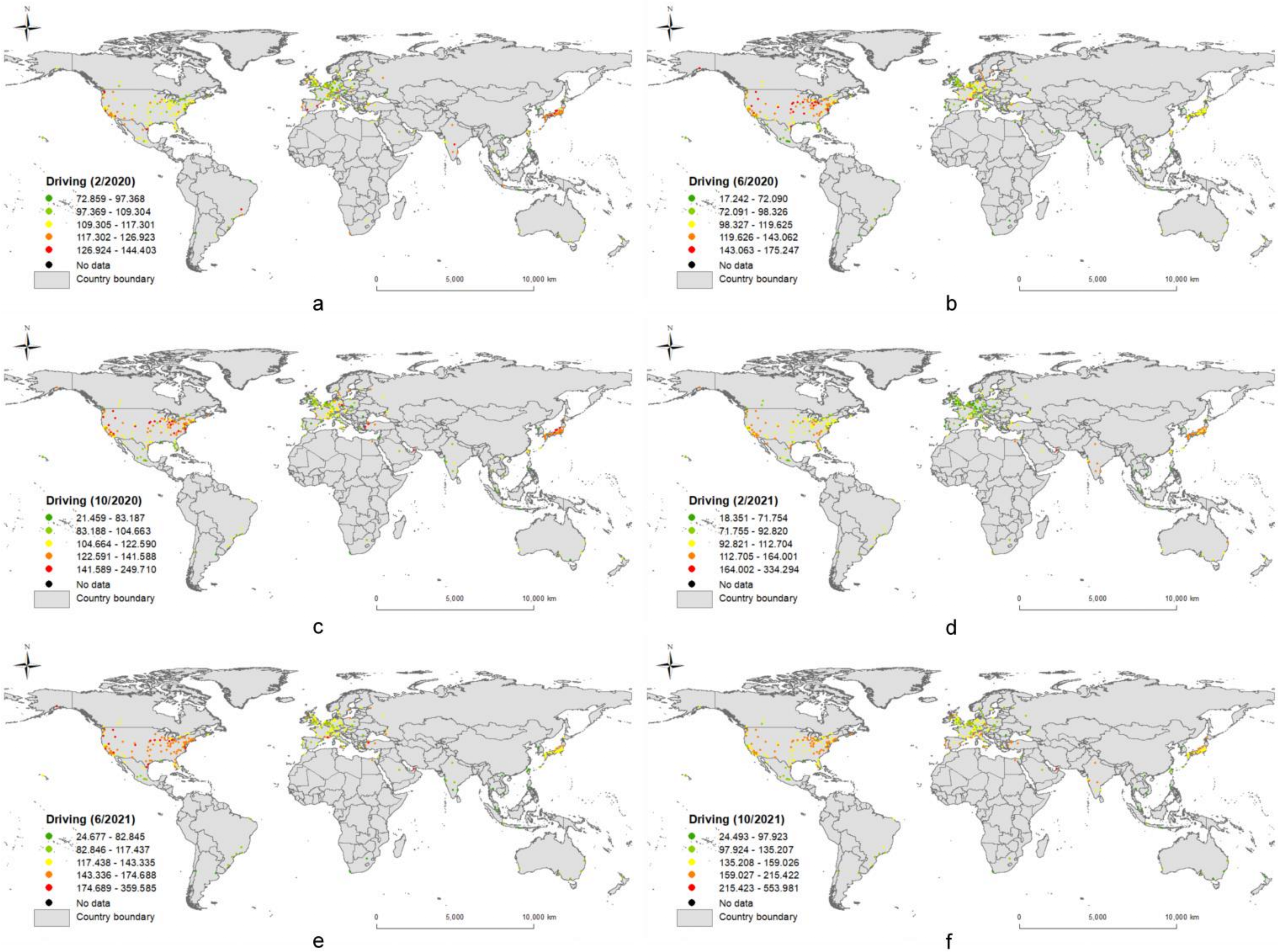

Figure. S6 The spatial distribution of driving index from 2020 to 2021: (a) 2/2020;(b) 6/2020; (c) 10/2020; (d) 2/2021; (e) 6/2021; (f) 10/2021

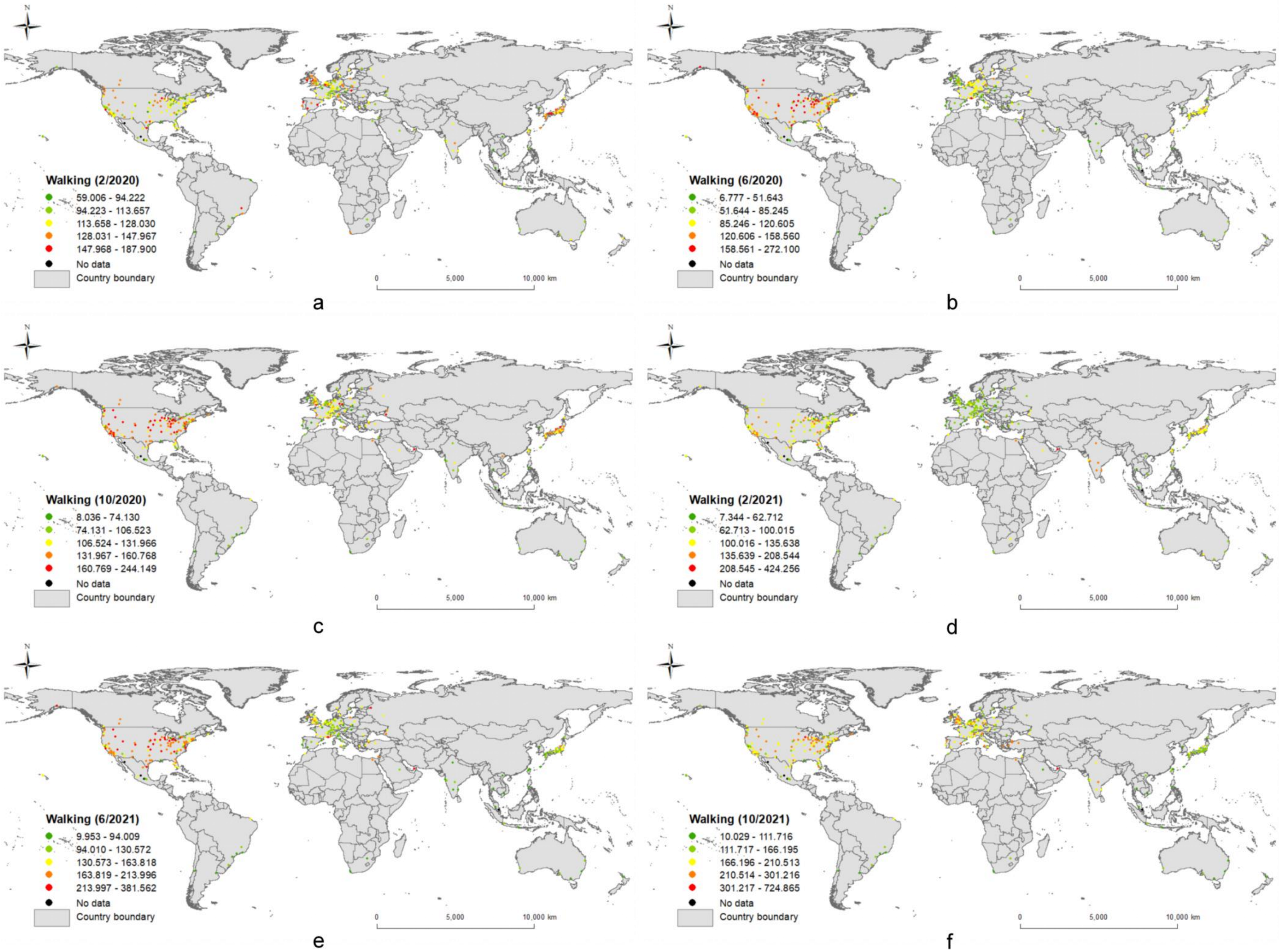

Figure. S7 The spatial distribution of walking index from 2020 to 2021: (a) 2/2020;(b) 6/2020; (c) 10/2020; (d) 2/2021; (e) 6/2021; (f) 10/2021

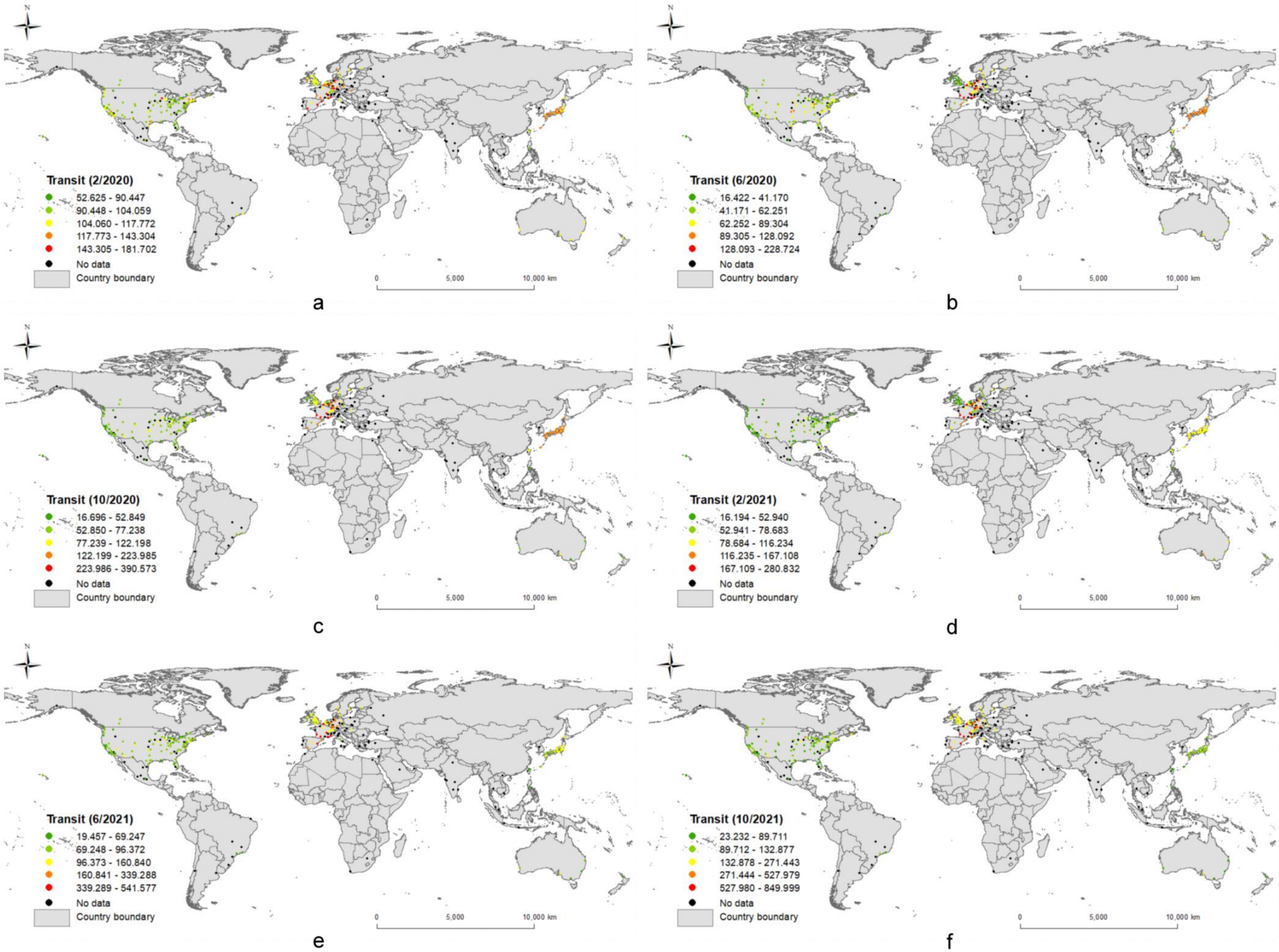

Figure. S8 The spatial distribution of transit index from 2020 to 2021: (a) 2/2020;(b) 6/2020; (c) 10/2020; (d) 2/2021; (e) 6/2021; (f) 10/2021

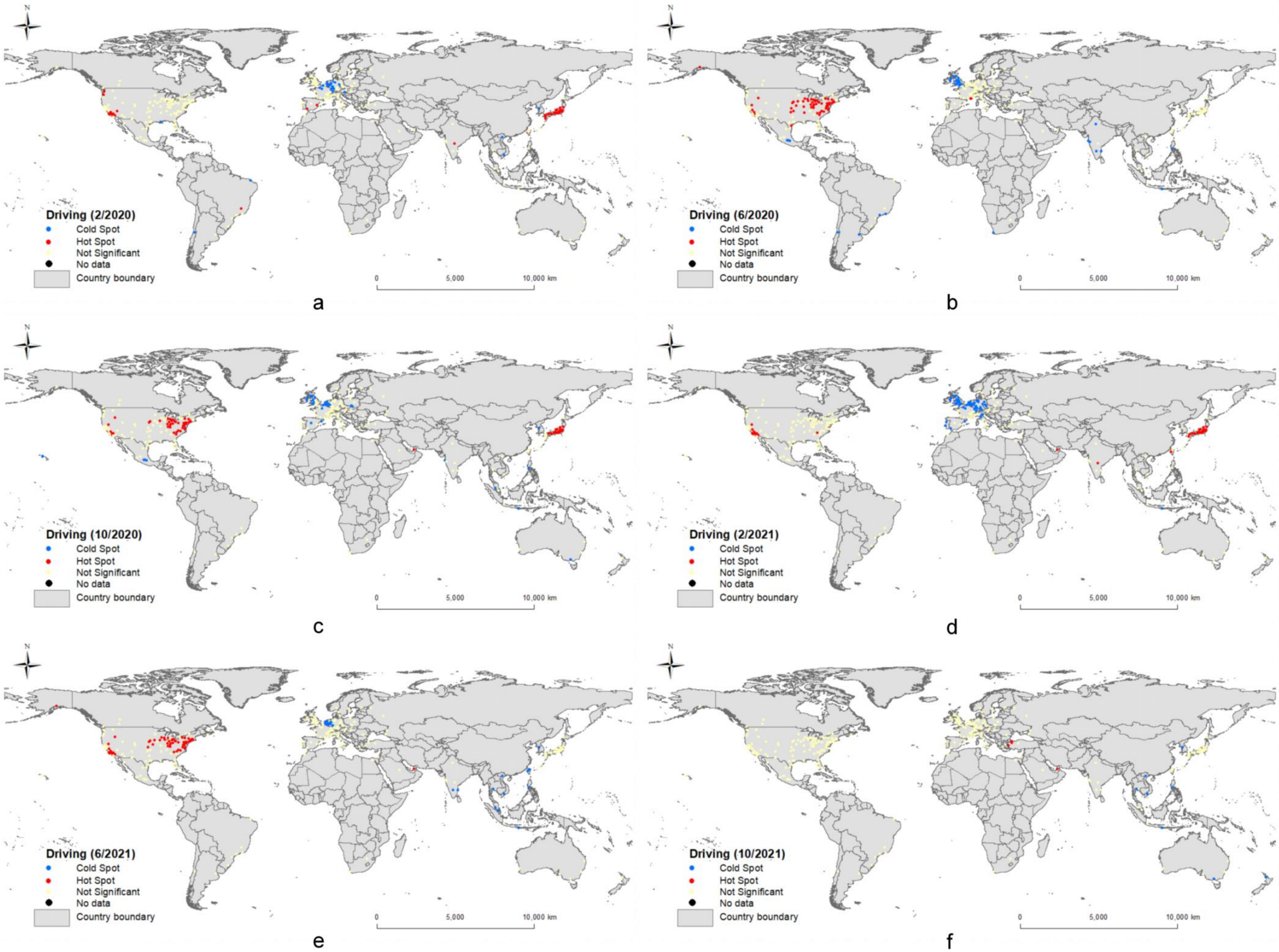

Fig. S9 Hot/cold spot map of driving index from 2020 to 2021: (a) 2/2020;(b) 6/2020; (c) 10/2020; (d) 2/2021; (e) 6/2021; (f) 10/2021

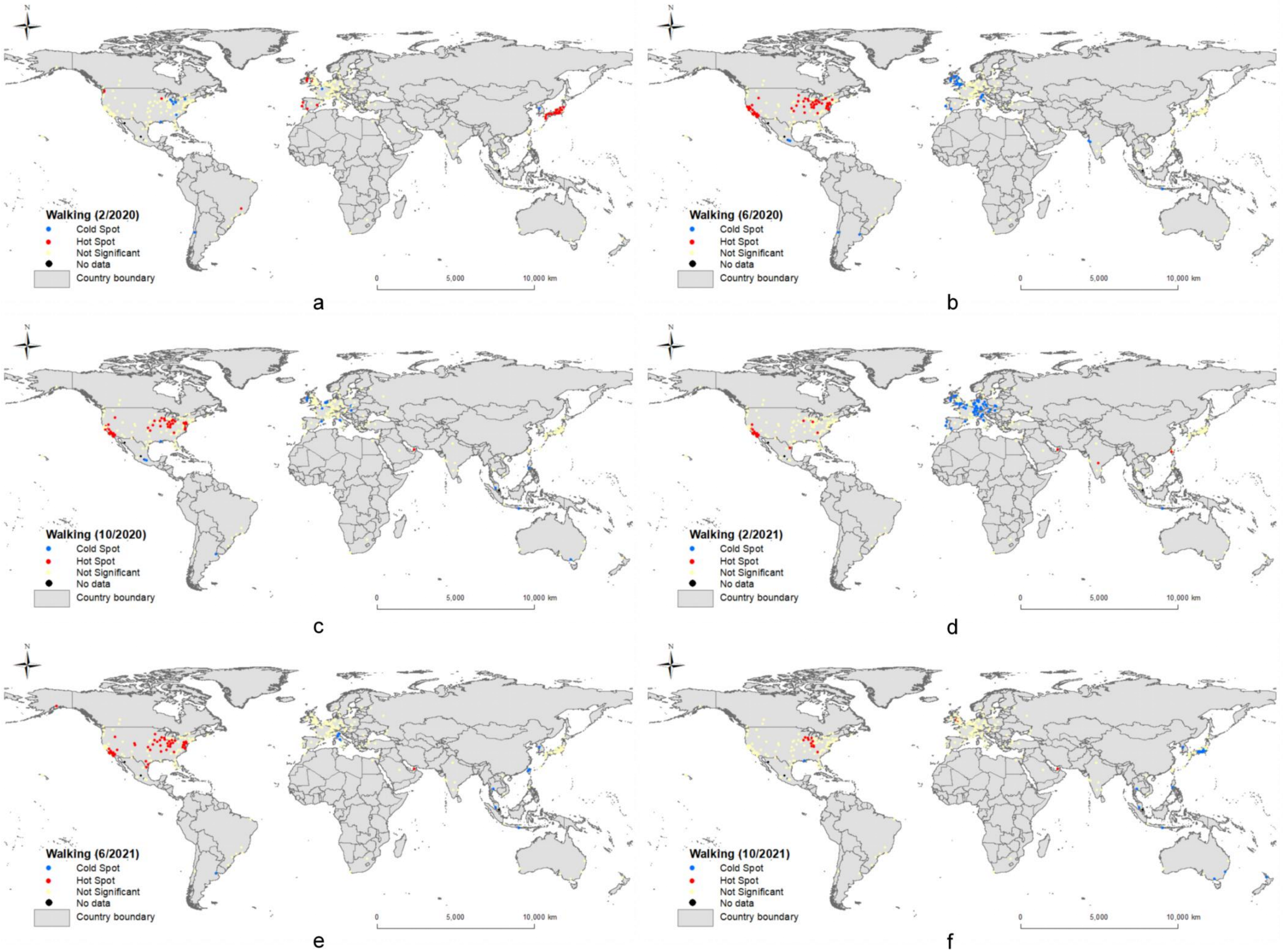

Fig. S10 Hot/cold spot map of the walking index from 2020 to 2021: (a) 2/2020;(b) 6/2020; (c) 10/2020; (d) 2/2021; (e) 6/2021; (f) 10/2021

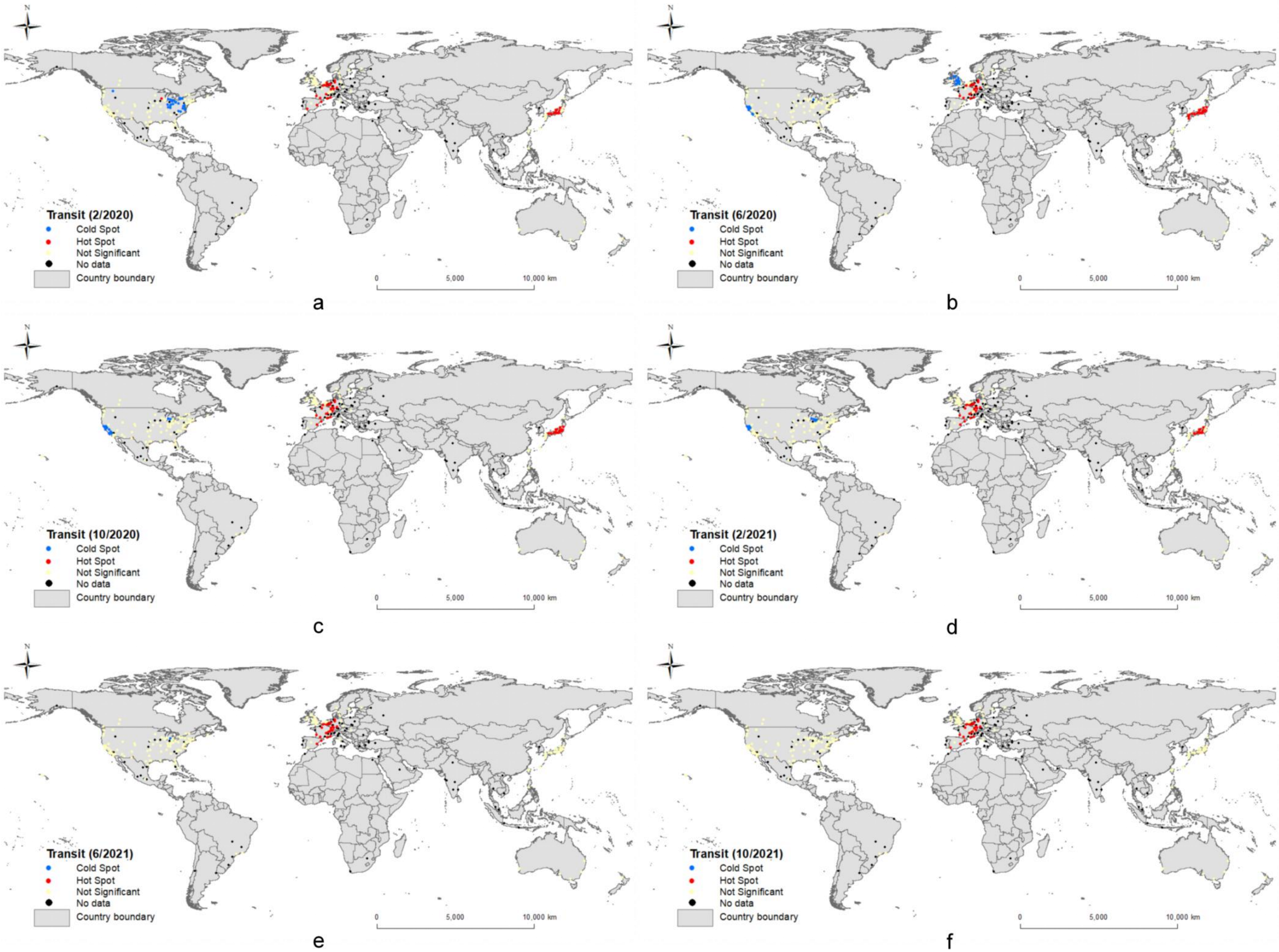

Fig. S11 Hot/cold spot map of transit index from 2020 to 2021: (a) 2/2020;(b) 6/2020; (c) 10/2020; (d) 2/2021; (e) 6/2021; (f) 10/2021

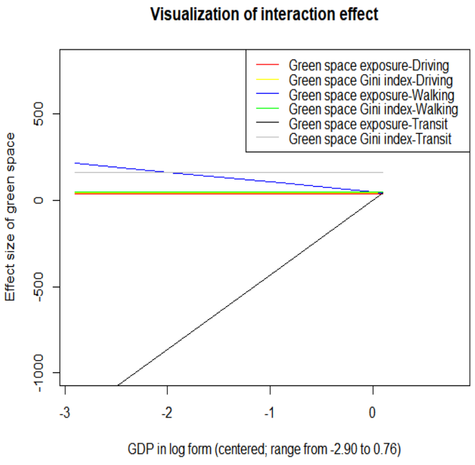
Visualization of interaction effect
Green space exposure-Driving
Green space Gini index-Driving
Green space exposure-Walking
Green space Gini index-Walking
Green space exposure-Transit
Green space Gini index-Transit
Effect size of green space
GDP in log form (centered; range from -2.90 to 0.76)

(a)

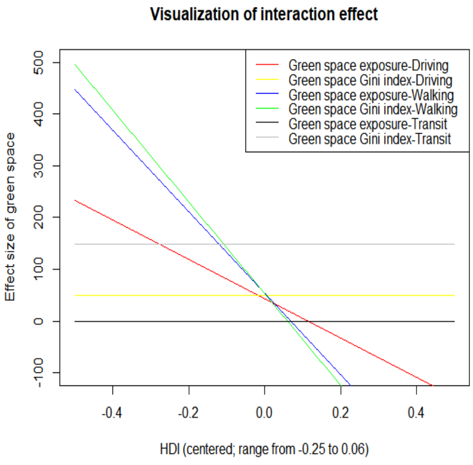
Visualization of interaction effect
Green space exposure-Driving
Green space Gini index-Driving
Green space exposure-Walking
Green space Gini index-Walking
Green space exposure-Transit
Green space Gini index-Transit
Effect size of green space
HDI (centered; range from -0.25 to 0.06)

(b)

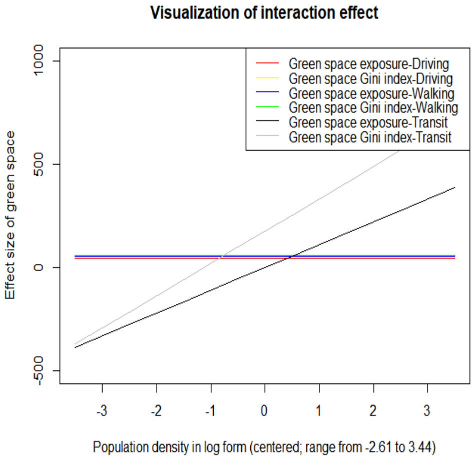
Visualization of interaction effect
Green space exposure-Driving
Green space Gini index-Driving
Green space exposure-Walking
Green space Gini index-Walking
Green space exposure-Transit
Green space Gini index-Transit
Effect size of green space
Population density in log form (centered; range from -2.61 to 3.44)

(c)

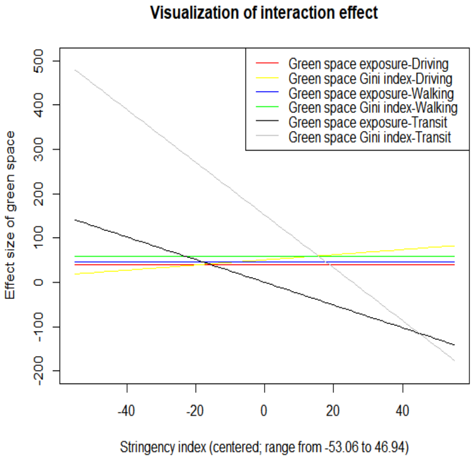
Visualization of interaction effect
Green space exposure-Driving
Green space Gini index-Driving
Green space exposure-Walking
Green space Gini index-Walking
Green space exposure-Transit
Green space Gini index-Transit
Effect size of green space
Stringency index (centered; range from -53.06 to 46.94)

(d)

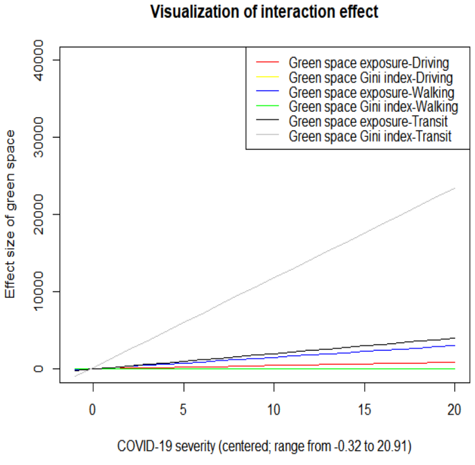
Visualization of interaction effect
Green space exposure-Driving
Green space Gini index-Driving
Green space exposure-Walking
Green space Gini index-Walking
Green space exposure-Transit
Green space Gini index-Transit
Effect size of green space
COVID-19 severity (centered; range from -0.32 to 20.91)

(e)

Fig S12. Visualization of interaction effect: (a) Model 5; (b) Model 6; (c) Model 7; (d) Model 8; (e) Model 9.

Table. S1 Descriptive statistics of all variables

	Theme	Scale	Variable Name (units)	Description	Source
Dependent variables	Environment	City	Green space exposure	Population-weighted green space coverage rate in 2020	https://datahub.hku.hk/projects/GreenExposure/140290
		City	Green space Gini index	Gini index of population-weighted green space coverage rate in 2020	https://datahub.hku.hk/projects/GreenExposure/140290
Dependent variables	Transportation	City	Driving index	Monthly Apple driving index from 2020 to 2021	COVID-19 - Mobility Trends Reports - Apple
		City	Walking index	Monthly Apple walking index from 2020 to 2021	COVID-20 - Mobility Trends Reports - Apple
		City	Public transit index	Monthly Apple public transit index from 2020 to 2021	COVID-21 - Mobility Trends Reports - Apple
Covariates	Demography	Country	Sex	The number of males for every 100 females in a population in 2021	Population Division of the Department of Economic and Social Affairs of the United Nations (https://population.un.org/wpp/)
		Country	Age	Median age (based on the model of the US Census Bureau) in 2020	The World Factbook (Central Intelligence Agency) (https://www.cia.gov/the-world-factbook/)
		City	Population density	The total population divided by the land area (persons/km2) in 2020	COVID-19 Open-Data (https://goo.gle/covid-19-open-data)
	Economy	Country	GDP	Gross domestic product per capita (US dollars) in 2020	The World Bank (https://www.worldbank.org/en/home)
		Country	HDI	Human development index in 2020. These variables measure average achievement in key dimensions of human development (life expectancy at birth, expected years of schooling, mean years of schooling, GNI per capita)	United Nations Development Program (https://hdr.undp.org/)
		City	Night light	The average brightness of night light. This variable can act as a proxy for the general level of infrastructure in 2020	Earth Observation Group (https://eogdata.mines.edu/products/vnl/)
	Environment	City	$PM_{2.5}$	Average annual $PM_{2.5}$ level (μg/m^3) in 2020	Atmospheric Composition Analysis Group (https://sites.wustl.edu/acag/)
	Policy	City	Stringency index	The index records the strictness of 'lockdown style' policies that primarily restrict people's behaviour. It is calculated using all ordinal containment and closure policy indicators, plus an indicator recording public information campaigns (from 2020 to 2021)	COVID-19 GOVERNMENT RESPONSE TRACKER (https://www.bsg.ox.ac.uk/research/covid-19-government-response-tracker)
	COVID-19 related	City	COVID-19 severity	The cumulative COVID-19 infection rate in 2020 and 2021	COVID-19 Open-Data (https://goo.gle/covid-19-open-data)
	Transportation	City	Motor City	Transportation-based city type (Medium to low density, high capacity, grid-based, road networks, medium railed transport)	https://www.thelancet.com/cms/10.1016/S2542-5196(19)30263-3/attachment/4ab590e0-e73f-406f-8432-3e01929010c2/mmc1.pdf
		City	High Transit	Transportation-based city type (Medium density, high capacity, formal road networks, high public transport)	https://www.thelancet.com/cms/10.1016/S2542-5196(19)30263-3/attachment/4ab590e0-e73f-406f-8432-3e01929010c2/mmc1.pdf
		City	Chequerboard	Transportation-based city type (High density, medium capacity mixed formal and informal road networks, medium public transport)	https://www.thelancet.com/cms/10.1016/S2542-5196(19)30263-3/attachment/4ab590e0-e73f-406f-8432-3e01929010c2/mmc1.pdf
		City	Informal	Transportation-based city type (Sparse, low-capacity informal road infrastructure, railed transport, low formal green space)	https://www.thelancet.com/cms/10.1016/S2542-5196(19)30263-3/attachment/4ab590e0-e73f-406f-8432-3e01929010c2/mmc1.pdf
		City	Cul de Sac	Transportation-based city type (Very high density, low capacity mixed formal and informal road networks, low mass transit)	https://www.thelancet.com/cms/10.1016/S2542-5196(19)30263-3/attachment/4ab590e0-e73f-406f-8432-3e01929010c2/mmc1.pdf

City	Large Block	Transportation-based city type (Medium density formal low and high-capacity road networks, medium railed transport)	https://www.thelancet.com/cms/10.1016/S2542-5196(19)30263-3/attachment/4ab590e0-e73f-406f-8432-3e01929010c2/mmc1.pdf
City	Irregular	Transportation-based city type (High green space, mixed formal and informal low and high-capacity road networks, low mass transit)	https://www.thelancet.com/cms/10.1016/S2542-5196(19)30263-3/attachment/4ab590e0-e73f-406f-8432-3e01929010c2/mmc1.pdf
City	Intense	Transportation-based city type (Very high density, mixed formal high capacity and informal road networks, high public transport)	https://www.thelancet.com/cms/10.1016/S2542-5196(19)30263-3/attachment/4ab590e0-e73f-406f-8432-3e01929010c2/mmc1.pdf

TableS2. Descriptive statistics of all variables

Variables	Mean (SD)/ Numbers (%)	Number of unique records
Green space exposure	0.43(0.21)	224
Green space Gini index	0.29(0.15)	224
High Transit (%)	73(32.59)	224
Chequerboard (%)	19(8.48)	224
Informal (%)	7(3.13)	224
Cul_de_Sac (%)	3(1.34)	224
Large Block (%)	10(4.46)	224
Irregular (%)	2(0.89)	224
Intense (%)	17(7.59)	224
Motor (%)	93(41.52)	224
Spring (%)	1344	5152(224 cities×23 months)
Summer (%)	1344	5152(224 cities×23 months)
Autumn (%)	1344	5152(224 cities×23 months)
Winter (%)	1120	5152(224 cities×23 months)
Sex	98.17(9.16)	224
Age	39.94(5.07)	224
GDP (in natural logarithm form)	47812.69(21514.69)	224
$PM_{2.5}$	11.83(10.27)	224
Night light	15.98(19.45)	224
HDI	0.90(0.07)	224
Population density (in natural logarithm form)	4112.88(6646.90)	224
Stringency index	53.06(18.58)	5152(224 cities×23 months)
COVID-19 severity	0.31(1.42)	448(224 cities×2 years)
Driving index	114.53(39.11)	5152(224 cities×23 months)
Walking index	124.96(58.01)	5129(223 cities×23 months)
Transit index	98.74(89.96)	3565(155 cities×23 months)

Table S3 Regression results for the multilevel model to examine the association between green space exposure and inequality and Apple mobility index

	Model 1: Driving Coef. (SE)	Model 2: Walking Coef. (SE)	Model 3: Transit Coef. (SE)
Green space exposure	38.52***(10.84)	46.82***(18.20)	75.62*(49.99)
Green space Gini index	50.48***(16.14)	58.88**(26.87)	162.07**(80.16)
High Transit (Ref: Motor)	-12.44***(3.91)	-28.42***(6.87)	8.70 (21.12)
Chequerboard (Ref: Motor)	-13.59**(5.86)	-36.08***(9.67)	14.34 (31.09)
Informal (Ref: Motor)	-30.81***(9.70)	-44.76***(16.17)	
Cul_de_Sac (Ref: Motor)	-37.20***(9.96)	-55.47***(16.92)	
Large Block (Ref: Motor)	-12.93**(5.63)	-14.49 (9.96)	-46.41 (33.99)
Intense (Ref: Motor)	-8.53 (8.01)	-32.75**(14.20)	-30.77 (49.15)

Irregular (Ref: Motor)	53.78***(16.15)	54.55**(26.14)	
Summer (Ref: Spring)	26.59***(1.09)	21.63***(1.58)	23.63***(2.81)
Autumn (Ref: Spring)	28.27***(1.11)	32.74***(1.61)	39.87***(2.85)
Winter (Ref: Spring)	9.33***(1.16)	6.93***(1.68)	-0.58 (2.94)
Sex	0.68***(0.17)	0.98***(0.28)	-3.77 (4.74)
Age	0.87*(0.47)	1.06 (0.79)	-0.81 (3.43)
GDP	4.21 (4.94)	-3.70 (8.16)	-53.17 (32.24)
$PM_{2.5}$	0.25*(0.15)	0.38 (0.25)	-3.53 (2.34)
Night light	0.06 (0.07)	-0.11 (0.11)	-0.23 (0.35)
HDI	-86.13 (73.61)	-44.98 (120.58)	792.80 (583.64)
Population density	-3.91**(1.50)	-4.71* (2.48)	10.98 (8.02)
Stringency index	-0.76***(0.03)	-1.18***(0.04)	-1.59***(0.07)
COVID-19 severity	-0.47**(0.22)	1.05***(0.32)	127.82***(6.96)

Coeff. = coefficient; SE = standard error; *$p < .10$, **$p < .05$, ***$p < .01$.

Table S4 Regression results for the multilevel model to examine the association between green space exposure and inequality, and Apple mobility index (moderation analysis)

	Model 4a: Driving Coef. (SE)	Model 4b: Walking Coef. (SE)	Model 4c: Transit Coef. (SE)
Green space exposure×Green space Gini index	37.49 (51.03)	32.86 (84.64)	143.46 (237.07)
Green space exposure	42.06***(11.87)	49.88**(19.94)	86.59 (53.26)
Green space Gini index	58.84***(19.69)	66.02**(32.67)	191.53**(93.78)
	Model 5a: Driving	Model 5b: Walking	Model 5c: Transit
Green space Gini index×GDP	-23.68 (16.34)	-39.30*(22.65)	-144.90 (240.93)
Green space exposure×GDP	-14.81 (13.36)	-56.44**(27.46)	431.80**(173.09)
Green space exposure	38.19***(11.89)	49.02**(19.05)	2.13 (52.89)
Green space Gini index	45.11***(17.02)	48.45**(24.04)	160.12**(79.13)
GDP	3.57 (5.08)	-4.74 (8.27)	-38.82 (31.90)
	Model 6a: Driving	Model 6b: Walking	Model 6c: Transit
Green space Gini index×HDI	-322.01 (236.88)	-883.84**(400.40)	-4343.72 (4194.41)
Green space exposure×HDI	-380.29** (190.51)	-786.35**(340.60)	4030.94 (2574.74)
Green space exposure	43.37***(11.38)	53.83***(18.65)	60.21 (49.29)
Green space Gini index	50.18***(16.42)	53.20**(27.10)	148.28**(77.95)
HDI	-124.83* (75.41)	-130.32 (123.23)	1691.24***(635.74)
	Model 7a: Driving	Model 7b: Walking	Model 7c: Transit
Green space Gini index×Population density	-6.57 (11.58)	5.44 (19.53)	156.03**(64.17)
Green space exposure×Population density	8.67 (9.79)	16.90 (16.42)	109.87**(48.70)
Green space exposure	45.06***(11.14)	52.81***(18.80)	81.28 (50.34)
Green space Gini index	53.24***(16.09)	59.49**(26.91)	175.47**(79.49)
Population density	-2.07 (1.70)	-2.92 (2.80)	15.11* (8.49)
	Model 8a: Driving	Model 8b: Walking	Model 8c: Transit
Green space Gini index×Stringency index	0.58**(0.28)	0.70*(0.40)	-5.95***(0.90)

Green space exposure×Stringency index	0.31 (0.23)	-0.02 (0.33)	-2.56***(0.62)
Green space exposure	38.70***(10.83)	46.27**(18.24)	71.16 (50.45)
Green space Gini index	50.64***(16.11)	58.74**(26.92)	151.73**(75.90)
Stringency index	-0.77***(0.03)	-1.20***(0.04)	-1.62***(0.07)
	Model 9a: Driving	Model 9b: Walking	Model 9c: Transit
Green space Gini index×COVID-19 severity	2.52 (2.81)	4.44 (4.02)	1161.44***(114.27)
Green space exposure×COVID-19 severity	43.48***(5.28)	148.71***(10.28)	201.64***(68.94)
Green space exposure	41.10***(10.98)	56.97***(18.58)	84.89*(49.71)
Green space Gini index	47.81***(16.21)	53.07*(27.36)	191.32**(79.78)
COVID-19 severity	13.54***(1.71)	49.81***(3.37)	146.87***(7.09)

All covariates were adjusted. Coeff. = coefficient; SE = standard error; *p < .10, **p < .05, ***p < .01.
